# Epidemiology of Carbapenem-Resistant *Acinetobacter baumannii* Bloodstream Infections in Poland: A Multi-Center Study of Mortality, Risk Factors and Drug Resistance

**DOI:** 10.3390/jcm15020527

**Published:** 2026-01-08

**Authors:** Agnieszka Kuncka, Patrycja Leśnik, Jarosław Janc, Katarzyna Dzierżanowska-Fangrat, Martyna Biała, Paulina Kołat-Brodecka, Natalia Słabisz

**Affiliations:** 1 Department of Anaesthesiology and Intensive Therapy, Mazovian Brodnowski Hospital, 03-242 Warsaw, Poland; akuncka@gmail.com; 2 Department of Microbiology, Wroclaw Medical University, 50-556 Wrocław, Poland; 3 Department of Emergency Medical Service, Faculty of Nursing and Midwifery, Wroclaw Medical University, 50-556 Wrocław, Poland; jarojanc@gmail.com; 4 Department of Clinical Microbiology and Immunology, Children’s Memorial Health Institute, 04-730 Warsaw, Poland; k.fangrat@ipczd.pl; 5 Department of Infectious Diseases, Liver Diseases and Acquired Immune Deficiences, Faculty of Medicine, Wroclaw Medical University, 51-149 Wrocław, Poland; biala.martyna@interia.pl; 6 Hospital Infection Prevention and Control Team, University Clinical Hospital of the Military Medical Academy-Central Veterans Hospital, 90-549 Lodz, Poland; paulinabrodecka@icloud.com; 7 Department of Preclinical Sciences, Pharmacology and Medical Diagnostics, Faculty of Medicine, Wroclaw University of Science and Technology, 50-370 Wrocław, Poland; 8 Department of Laboratory Diagnostics, 4th Military Clinical Hospital, 50-981 Wrocław, Poland

**Keywords:** *Acinetobacter*, CRAB, bloodstream infections, mortality, resistance, antibiotics, risk factors

## Abstract

**Background**: *Acinetobacter baumannii* (AB), particularly carbapenem-resistant strains (CRAB), is a major cause of difficult-to-treat infections associated with substantial mortality. Contemporary data from Central and Eastern Europe remain scarce. We aimed to characterize the epidemiology, clinical features, and survival of patients with AB bloodstream infection in a multicenter Polish cohort. **Methods**: We conducted a retrospective multicenter study including consecutive adults with microbiologically confirmed AB bloodstream infection. Clinical and demographic data, comorbidities, infection origin, and antimicrobial treatments were collected. Outcomes included all-cause in-hospital mortality and infection-attributed mortality. Survival was assessed using Kaplan–Meier curves and log-rank tests, while factors associated with death were examined with univariable and multivariable Cox regression. **Results**: Among 245 patients with CRAB bloodstream infection, overall mortality was 69.4%, and infection-attributed mortality reached 51.8%. Most infections (75.1%) were hospital-acquired. In univariable analyses, male sex (HR = 0.66; *p* = 0.008) and colistin-based therapy (HR = 0.71; *p* = 0.037) were associated with improved survival. Conversely, hospital-acquired infection (HR = 0.43; *p* < 0.001) and acute kidney injury (HR = 1.40; *p* = 0.038) were linked to higher mortality. In the multivariable model, male sex remained protective (HR = 0.61; *p* = 0.006), while hospital-acquired infection (HR = 0.35; *p* < 0.001) and COVID-19 (HR = 1.64; *p* = 0.049) independently predicted death. After adjustment, no other comorbidities or antimicrobial regimens showed significant associations. **Conclusions**: In this multicenter cohort of patients with CRAB bloodstream infection, mortality remained extremely high. Hospital-acquired infection, acute kidney injury, and COVID-19 were strong independent predictors of poor outcomes, whereas male sex was associated with better survival. Although colistin-containing therapy appeared beneficial in univariable analysis, this effect did not persist after adjustment, underscoring potential confounding. These findings highlight the urgent need for early recognition, optimized antimicrobial strategies, and prevention of healthcare-associated spread to improve outcomes in CRAB bacteremia.

## 1. Introduction

*Acinetobacter baumannii* has become one of the most challenging pathogens in modern critical care settings. This opportunistic, Gram-negative coccobacillus is especially problematic in intensive care units (ICUs), where vulnerable patients—those with prolonged hospital stays, invasive devices, and exposure to broad-spectrum antibiotics—are at an elevated risk [1]. Once regarded as a relatively low-virulence organism, *A. baumannii* has evolved into a significant cause of healthcare-associated infections, including ventilator-associated pneumonia, bloodstream infections, urinary tract infections, and wound infections [2]. The clinical concerns surrounding *A. baumannii* are heightened by its remarkable ability to survive in hospital environments and its tendency to develop multidrug resistance (MDR). Mechanisms such as efflux pumps, β-lactamase production, and biofilm formation have contributed to the organism’s persistence and resistance to multiple classes of antibiotics. In many regions, carbapenem-resistant *A. baumannii* (CRAB) has become widespread, leaving clinicians with limited and often toxic treatment options, such as polymyxins or tigecycline [3,4,5]. Infections caused by *A. baumannii* in the ICU are linked to significant morbidity, prolonged hospital stays, increased healthcare costs, and elevated mortality rates. The global rise of MDR and extensively drug-resistant (XDR) strains highlights the urgent need for improved prevention strategies, timely diagnoses, and effective treatment protocols. Understanding the epidemiology, risk factors, resistance mechanisms, and clinical outcomes of *A. baumannii* infections in critically ill patients is essential for guiding infection control measures and optimizing patient management [6,7]. However, data on the burden of AB bloodstream infections in Central and Eastern Europe remain limited. This study aims to characterize the epidemiology, clinical features, and survival of patients with AB bloodstream infection in a multi-center Polish cohort.

## 2. Materials and Methods

### 2.1. Study Design

Data analysis began in early 2025 and was entirely retrospective. This multicenter study included 245 patients hospitalized between 2015 and 2023 at three Polish hospitals: Mazowiecki Brodnowski Hospital in Warsaw (*n* = 100), the University Clinical Hospital No. 2 of the Medical University of Łódź (*n* = 89), and the 4th Military Clinical Hospital in Wrocław (*n* = 56). All patients had at least one blood culture positive for carbapenem-resistant *Acinetobacter baumannii* (CRAB). The analyzed cases did not originate from recognized outbreak situations and represented epidemiologically unrelated, sporadic infections. Only episodes of *A. baumannii* bloodstream infection (BSI) were analyzed, as the inclusion criterion was a positive blood culture for this pathogen. Inflammatory and clinical parameters were assessed at the time of blood culture collection. For each case, an attempt was made to determine whether the bacteremia originated from a primary infectious focus and, if so, to identify its probable source based on clinical, microbiological, and imaging data when available.

### 2.2. Data Collection

Demographic data such as age, sex, and comorbidities (diabetes, chronic kidney disease, chronic obstructive pulmonary disease, chronic cardiovascular disease, and cancer), overweight (BMI ≥ 25), as well as other risk factors for infection including the presence of mechanical ventilation, intravascular lines, previous surgical procedures, parenteral nutrition, artificial valves or prosthetic implants, and coexisting COVID-19 infection were obtained from the hospital database, including information on patient outcomes taking into account the patients’ medical histories. Selected hematological and biochemical parameters (white blood cell count [WBC; ×10^3^/µL], neutrophil count [NEUTR; ×10^3^/µL], serum C-reactive protein level [CRP; mg/L], serum creatinine level [CRE; mg/dL], serum urea level [UREA; mg/dL], and serum procalcitonin level [PCT; ng/mL]) from the same day as the collection of the first blood sample yielding a positive culture were extracted from the electronic laboratory database.

### 2.3. Definitions

Following the *Surviving Sepsis Campaign* guidelines [8], in cases of suspected sepsis or septic shock, two to three sets of blood cultures should be collected before initiating antimicrobial therapy, using bottles suitable for both aerobic and anaerobic organisms. Blood culture testing was indicated in patients with suspected bacterial endocarditis, indwelling catheters, or fever > 39.4 °C. When fever exceeded 38.3 °C and was accompanied by at least two additional risk factors (age > 65 years, chills, vomiting, systolic blood pressure < 90 mmHg, leukocytosis, or serum creatinine > 2 mg/dL), cultures were also obtained. Empirical antibiotic therapy was initiated within one hour of diagnosis, and in cases with suspected MRSA or MDRO bloodstream infection, broad-spectrum agents were used accordingly.

A bloodstream infection (BSI) was defined as the recovery of *Acinetobacter baumannii* from at least one blood culture. Obesity was defined as a body mass index (BMI) ≥ 30.0 kg/m^2^, calculated as weight (kg) divided by height (m) squared. Acute kidney injury (AKI) was defined as any of the following: an increase in serum creatinine (SCr) by ≥0.3 mg/dL (≥26.5 µmol/L) within 48 h; or an increase in SCr to ≥1.5 × baseline (known or presumed to have occurred within the prior 7 days); or urine output < 0.5 mL/kg/h for ≥6 h.

Infection-attributed mortality was defined as death directly resulting from the infectious process or its complications, as determined by the treating physicians based on clinical, microbiological, and radiological findings.

Community-acquired infection was defined as bacteremia diagnosed within the first 48 h of hospital admission in patients without a history of hospitalization or invasive medical procedures within the preceding 90 days. Hospital-acquired infection was defined as bacteremia occurring ≥48 h after hospital admission or in patients with recent healthcare exposure, including prior hospitalization, invasive procedures, or the presence of indwelling medical devices. Classification was based on a retrospective review of clinical records and microbiological data.

### 2.4. Applied Therapeutic Regimens

Antibiotic treatment options for carbapenem-resistant *Acinetobacter baumannii* (CRAB) infections remain severely limited. Although the β-lactam/β-lactamase inhibitor combination sulbactam/durlobactam received FDA approval in 2023, it is currently unavailable in Poland. Consequently, therapeutic management at the participating centers relied on older combination regimens. The most commonly used treatment approaches included ampicillin–sulbactam combined with colistin, colistin in combination with an aminoglycoside and a carbapenem, or ampicillin–sulbactam with tigecycline. These regimens are frequently associated with limited clinical efficacy and a high risk of adverse effects, particularly colistin-related nephrotoxicity.

Cefiderocol represents a potential therapeutic alternative; however, its use remains restricted due to limited availability and substantial cost. In addition, concerns regarding its clinical effectiveness persist. The CREDIBLE-CR study reported increased mortality among patients treated with cefiderocol for CRAB infections, which was attributed to heteroresistance and the emergence of resistance during therapy [9]. These findings underscore the ongoing challenges in the treatment of CRAB infections and highlight the urgent need for improved access to novel antimicrobial agents and optimized therapeutic strategies.

### 2.5. Statistics

All analyses were performed using Python (v3.11) with the *pandas*, *scipy.stats*, *lifelines*, and *matplotlib* libraries. Descriptive statistics were calculated for all study variables. Quantitative variables (e.g., age, WBC, CRP, PCT, creatinine, length of stay, ICU days) were expressed as mean ($\bar{x}$), standard deviation (SD), median (Me), interquartile range (Q1–Q3), and minimum and maximum values. Qualitative variables (e.g., sex, comorbidities, antibiotic resistance, infection source) were summarized as absolute numbers (*n*) and percentages (%). Survival analyses were carried out using Kaplan–Meier survival curves, with the log-rank test applied to compare survival distributions between groups. Univariate Cox proportional hazards regression analyses were then conducted to identify clinical and microbiological factors associated with mortality. Results are presented as hazard ratios (HR) with 95% confidence intervals (CI) and *p*-values. For categorical predictors, one-hot encoding was applied, and the first category served as the reference level. A *p*-value < 0.05 was considered statistically significant.

### 2.6. Ethics

The study protocol was approved by the Bioethics Committee of the Lower Silesian Chamber of Physicians in Wroclaw, Poland (approval no. 5/BNR/2023). Confidentiality and privacy regarding personal, laboratory, and clinical information were strictly maintained. The study was conducted in compliance with the principles of the Declaration of Helsinki and Good Clinical Practice guidelines.

## 3. Results

### 3.1. Patient Characteristics

Among 245 patients with *Acinetobacter baumannii* bacteremia, 55.5% were male. Most admissions occurred in Internal Medicine wards and the ICU. Cardiovascular disease, diabetes, and chronic kidney disease were the most frequent comorbidities. Over one-third of patients required ICU stay and mechanical ventilation. The majority of infections were hospital-acquired (75.1%), with pulmonary involvement being the most common presumed source of bacteremia. Overall mortality was high (69.4%), with infection-attributed mortality of 51.8%. Patients were elderly, exhibited marked systemic inflammation, and experienced prolonged hospitalization (Table 1 and Table 2). The median length of hospital stay was 19 days (IQR: 1-381).

Among the study population, 161 patients received colistin-based therapy. Of these, 19 patients were treated with colistin monotherapy, 77 received a combination of colistin and meropenem, and 36 were treated with colistin combined with ampicillin-sulbactam. In 29 patients, a triple-drug regimen consisting of colistin, tigecycline, and amikacin was administered.

The remaining 84 patients received antibiotic regimens that did not include colistin. Among these patients, 42 were treated with a combination of amikacin and meropenem, 21 received amikacin combined with ampicillin-sulbactam, 16 were treated with amikacin and tigecycline, and 5 patients received meropenem in combination with tigecycline.

### 3.2. Antimicrobial Susceptibility of the Isolates

As only patients infected with carbapenem-resistant *Acinetobacter baumannii* (CRAB) were included in the study, all 245 isolates were resistant to imipenem and meropenem. Colistin demonstrated the highest in vitro activity, with 96% of isolates remaining susceptible. In contrast, susceptibility to gentamicin was observed in only 41% of isolates. A high rate of resistance to ciprofloxacin was noted, as 93% of the strains were classified as resistant. Susceptibility to tigecycline was observed in 77% of isolates, whereas only 36% of strains were susceptible to the ampicillin–sulbactam combination.

### 3.3. Survival Analysis

**Figure 1:**
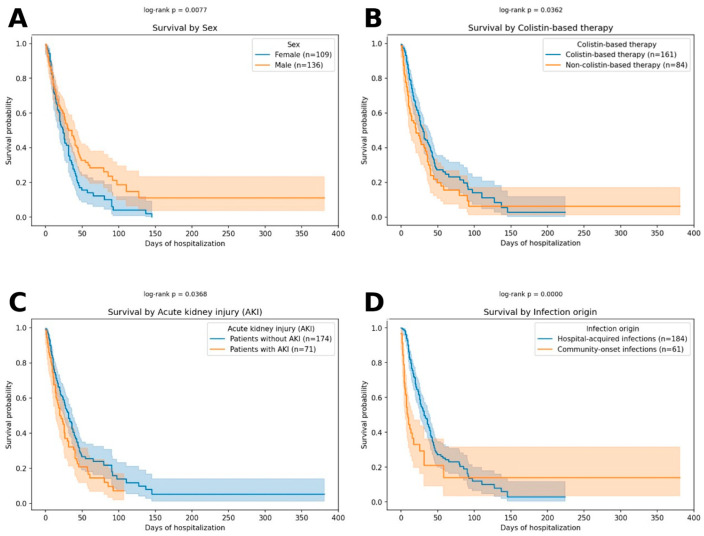
Kaplan–Meier overall survival in patients with *Acinetobacter baumannii* infection according to selected clinical factors. (**A**) Survival by sex. (**B**) Survival by colistin-based therapy. (**C**) Survival according to the presence of acute kidney injury (AKI). (**D**) Survival by infection origin (community-acquired vs. hospital-acquired). Solid lines represent Kaplan–Meier survival estimates, and shaded areas indicate 95% confidence intervals. Time is expressed as days since the index date. The event of interest was all-cause in-hospital death; observations were censored at hospital discharge or at the end of follow-up. Survival differences between groups were assessed using the log-rank test.

In the univariate Kaplan–Meier survival analysis, significant differences were observed for several clinical factors (Figure 1). Survival probability was higher in males compared with females (log-rank *p* = 0.008). Use of colistin was associated with improved survival, particularly in the early observation period (*p* = 0.036). In contrast, patients who developed acute kidney injury (AKI) had significantly worse outcomes compared with those without AKI (*p* = 0.037). Infection origin was also strongly associated with survival (*p* < 0.001): hospital-acquired infections were linked with higher survival compared to community-acquired cases.

### 3.4. Infection Origin and Clinical Features

Comparison of clinical characteristics between community- and hospital-acquired infections is presented in Table 3. Diabetes was significantly more frequent among patients with hospital-acquired infections (34.8% vs. 18.0%; *p* = 0.021). Vascular line use was also higher in this group (18.5% vs. 3.3%; *p* = 0.007). COVID-19 occurred exclusively in patients with hospital-acquired infections (15.2% vs. 0%; *p* = 0.003).

### 3.5. Cox Regression Analysis

In univariate Cox regression analysis, several demographic, clinical, and therapeutic factors were significantly associated with in-hospital mortality. Male sex was associated with a lower risk of death compared with females (HR = 0.66; 95% CI: 0.49–0.90; *p* = 0.008). Higher serum creatinine levels at diagnosis were associated with increased mortality risk (HR = 1.29; 95% CI: 1.14–1.45; *p* < 0.001), as was the presence of acute kidney injury (AKI) (HR = 1.40; 95% CI: 1.02–1.93; *p* = 0.038). Hospital-acquired infections were associated with significantly worse outcomes compared with community-acquired infections (*p* < 0.001). In contrast, colistin-based therapy was associated with a reduced risk of death (HR = 0.71; 95% CI: 0.52–0.98; *p* = 0.037). Detailed results are presented in Table 4.

In the multivariate Cox regression model, variables with a *p*-value < 0.30 in the univariate analysis, as well as clinically relevant factors (age, AKI, obesity, COPD, diabetes, chronic kidney disease, mechanical ventilation, use of colistin, and infection origin), were included. Male sex remained significantly associated with a lower risk of death (HR = 0.61; 95% CI: 0.43–0.87; *p* = 0.006), consistent with the findings of the univariate analysis. In contrast, hospital-acquired infections continued to be significantly associated with a higher risk of death compared with community-acquired infections (HR = 0.35; 95% CI: 0.23–0.52; *p* < 0.001—the lower HR for the reference category confirms worse prognosis for hospital-acquired infections). The presence of COVID-19 was also an independent predictor of poorer outcomes (HR = 1.64; 95% CI: 1.00–2.68; *p* = 0.049). Immunosuppression showed a trend toward increased mortality risk, although at the borderline of statistical significance (HR = 1.72; 95% CI: 0.99–2.98; *p* = 0.052). A similar trend was observed for AKI (HR = 1.59; *p* = 0.051), suggesting a potential impact of acute kidney injury on mortality, although the result did not reach the conventional significance threshold. In contrast, treatment-related variables (including colistin, gentamicin, amikacin, and fluoroquinolones) did not show a significant effect on mortality after adjustment for potential confounders.

## 4. Discussion

In this multicenter cohort of adults with *Acinetobacter baumannii* (AB) isolated from blood cultures, we observed a concerningly high mortality rate, with an overall rate of 69.4% and an infection-attributed rate of 51.8%. Survival rates varied across different clinical strata: they were higher in males compared to females, increased among patients who received colistin (based on univariable analysis), decreased in those who developed acute kidney injury (AKI), and were significantly higher for community-acquired infections compared with hospital-acquired cases. Pulmonary involvement was predominant, and device-related infection sources were common, consistent with the profile of older, comorbid inpatients with significant ICU exposure. Our crude mortality rate exceeds that reported in many single-center studies. Yet, it aligns with the broader ranges noted for AB bacteremia and severe AB infections, where short-term mortality rates typically fall between 30% and 70%—often higher among cases of carbapenem-resistant *Acinetobacter baumannii* (CRAB) [10,11]. Regionally, there is substantial resistance pressure in parts of Europe, particularly due to the rising carbapenem resistance in *Acinetobacter* species from 2017 to 2021, which has limited effective treatment options. Furthermore, the 2024 WHO Bacterial Priority Pathogens List continues to categorize carbapenem-resistant *A. baumannii* as a “critical priority,” highlighting the global urgency of the situation [12]. Polish and regional reports similarly emphasize the dissemination of CRAB and its associated adverse outcomes [13]. Interestingly, we also found that survival rates were better in males than in females. Most antibiotic (AB) studies have not consistently identified sex differences after multivariable adjustments, suggesting that our findings may reflect residual confounding factors, such as illness severity, treatment selection, or the differential site of infection. Current AB bacteremia cohorts emphasize severity of illness and the promptness of active therapy as the primary determinants of patient outcomes [14,15]. In our cohort, colistin was administered solely as part of combination regimens, which included a carbapenem, ampicillin-sulbactam, or an aminoglycoside. Thus, the lower mortality associated with colistin exposure in univariable models should be understood as linked to colistin-containing combination therapy rather than colistin monotherapy. This distinction is crucial because the advantages of colistin and carbapenem combinations over colistin alone have shown inconsistent results in randomized trials and meta-analyses. In contrast, sulbactam-based strategies are considered the most biochemically plausible partner against *Acinetobacter baumannii* due to sulbactam’s intrinsic PBP binding capabilities. Contemporary guidelines emphasize the use of sulbactam-durlobactam (with a carbapenem background) for treating carbapenem-resistant *Acinetobacter baumannii* (CRAB) when available; otherwise, high-dose ampicillin-sulbactam-containing regimens or cefiderocol are recommended. Routine use of colistin monotherapy is discouraged due to its nephrotoxicity and uncertain survival benefit. Our observed “colistin effect” likely reflects confounding factors related to treatment selection (e.g., the use of sulbactam-containing combinations) and the era of access to newer agents. Currently, colistin remains the first-line treatment for carbapenem-resistant *Acinetobacter baumannii* (CRAB). In 2025, Katip et al. identified a correlation between higher total colistin dosages and reduced mortality risk [16]. This conclusion is further bolstered by research conducted by Arrayasillapatorn et al. [17]. It is imperative to utilize elevated doses of antibiotics to effectively treat multidrug-resistant (MDR) infections, as optimal dosing is associated with improved rates of cure. Accordingly, the signal should be interpreted as hypothesis-generating until further analyses can differentiate between colistin combined with carbapenem, colistin with ampicillin-sulbactam, and colistin plus aminoglycosides compared to non-colistin counterparts, while also addressing potential immortal-time and indication biases [18,19,20,21,22]. Acute kidney injury (AKI) was prevalent (29%) and was independently linked to higher mortality rates in univariable Cox analysis. This finding aligns with substantial literature indicating that sepsis-associated AKI carries a significant mortality burden and results in prolonged hospitalization [23,24]. Mechanistically, the synergistic nephrotoxicity resulting from the combination of aminoglycosides and colistin, along with hemodynamic instability, may heighten the risk in cases of *A. baumannii* sepsis. Hospital-acquired infections in our cohort were associated with a higher hazard of death compared to community-onset cases. This finding aligns with most previous studies on Gram-negative bacteremia and *A. baumannii* infections, which have consistently reported increased mortality among hospital-acquired cases [25,26].

### Limitations of the Study

This study is retrospective and limited to AB bacteremia, so findings may not generalize to other sites. Infection origin may be misclassified (many “community-onset” cases had prior healthcare exposure). Microbiology and severity data were limited (no uniform MIC/genotyping; no standardized SOFA/APACHE), and treatment was nonrandomized colistin used only in combinations with limited timing data, so confounding by indication and possible immortal-time bias remain. We mainly report univariable associations and in-hospital mortality only.

## 5. Conclusions

In this cohort of adults with *Acinetobacter baumannii* bacteremia, mortality was high, reflecting the substantial clinical burden of this pathogen. Outcomes were influenced by host factors, treatment, and infection characteristics; colistin-based therapy was associated with lower mortality, although this finding should be interpreted cautiously due to potential confounding. Acute kidney injury was strongly associated with poor outcomes, underscoring the importance of renal monitoring during treatment. Overall, these results highlight the ongoing challenges in managing *A. baumannii* bloodstream infections in settings with high antimicrobial resistance and emphasize the need for optimized antimicrobial stewardship, improved access to effective therapies, and further research to identify optimal treatment strategies.

## Figures and Tables

**Table 1:** Descriptive statistics for qualitative variables.

	Category	*n*	%
Sex	M	136	55.5
	F	109	44.5
Ward	Internal	111	45.3
	Intensive care	55	22.4
	Cardiology	31	12.7
	Neurology	30	12.2
	Surgery	18	7.3
	Missing	2	0.8
Obesity		18	7.3
Age > 75 y.		112	45.7
Diabetes		75	30.6
Cancer		57	23.3
Immunosuppression		22	9
TPN		9	3.7
Surgical procedure		2	0.8
Vascular devices		36	14.7
Mechanical ventilation		91	37.1
Chronic kidney disease		75	30.6
Cardiovascular diseases		134	54.7
COPD		43	17.6
Implants/prosthetics		21	8.6
COVID-19		28	11.4
Probable primary source of bacteremia	Pneumonia	136	55.5
	UTI	22	9
	SSI	7	2.9
	IAI	14	5.7
	SST	9	3.7
	Meningitis	4	1.6
	CVC	24	9.8
	Endocarditis	2	0.8
	Spondylodiscitis	2	0.8
	Unknown source	70	28.6
Death		170	69.4
Death due to infection		127	51.8
ICU stay		91	37.1
AKI		71	29
HAI		184	75.1

TPN—total parenteral nutrition, COPD—chronic obstructive pulmonary disease, UTI—urinary tract infection, SSI—surgical site infection, IAI—intra-abdominal infection, SST—skin and soft tissue infection, CVC—central venous catheter infection, AKI—acute kidney injury, HAI—hospital-acquired infection.

**Table 2:** Descriptive statistics for quantitative variables.

Variable	Mean $(\bar{x}$)	SD	Median (Me)	Q1	Q3	Min	Max
Age	72.92	15.2	74	66	85	20	99
WBC; ×10^3^/µL	12.88	8.29	11.74	7.54	16.58	0.07	48.4
NEUTR; ×10^3^/µL	79.51	16.77	84.2	75.18	90.5	0.6	98.3
CRP; mg/L	177.01	113.2	161	93.5	238.1	1	582.3
PCT; ng/mL	8.3	17.18	2.2	0.48	7.43	0.02	181
Creatinine; mg/dL	1.65	1.24	1.18	0.79	2.18	0.15	7.03
Length of stay (days)	30.02	37.05	19	10	39	0	381
ICU days	6.41	14.21	0	0	7	0	89

**Table 3:** Comparative analysis of clinical and demographic characteristics by infection origin—CAI (community-acquired infection) vs. HAI (hospital-acquired infection).

Variable		*n* (%) CAI	*n* (%) HAI	*p* (Chi^2^)
Sex	M	23 (37.7%)	86 (46.7%)	0.279
	F	38 (62.3%)	98 (53.3%)	
Obesity		2 (3.3%)	16 (8.7%)	0.262
Age > 75 y.		30 (49.2%)	82 (44.6%)	0.632
Diabetes		11 (18.0%)	64 (34.8%)	0.021
Cancer		9 (14.8%)	48 (26.1%)	0.101
Immunosuppression		5 (8.2%)	17 (9.2%)	1
TPN		0 (0.0%)	9 (4.9%)	0.172
Surgical procedure		0 (0.0%)	2 (1.1%)	1
Vascular devices		2 (3.3%)	34 (18.5%)	0.007
Mechanical ventilation		19 (31.1%)	72 (39.1%)	0.334
Chronic kidney disease		17 (27.9%)	58 (31.5%)	0.707
Cardiovascular diseases		36 (59.0%)	98 (53.3%)	0.526
COPD		11 (18.0%)	32 (17.4%)	1
Implants/prosthetics		5 (8.2%)	16 (8.7%)	1
COVID-19		0 (0.0%)	28 (15.2%)	0.003

TPN—total parenteral nutrition, COPD—chronic obstructive pulmonary disease, HAI—hospital-acquired infection.

**Table 4:** Univariable Cox regression analysis for mortality.

Variable		Hazard Ratio	95% CI Lower	95% CI Upper	*p*-Value
Sex		0.66	0.49	0.90	0.008
Obesity		1.01	1.00	1.02	0.179
Age > 75 y.		1.11	0.82	1.51	0.489
Diabetes		0.95	0.84	1.07	0.423
Cancer		1.14	0.63	2.05	0.665
Immunosuppression		0.85	0.61	1.18	0.33
TPN		0.89	0.62	1.28	0.524
Surgical procedure		1.48	0.91	2.42	0.114
Vascular devices		0.87	0.40	1.85	0.709
Mechanical ventilation		0.44	0.06	3.18	0.419
Chronic kidney disease		0.80	0.52	1.23	0.309
Cardiovascular diseases		0.92	0.68	1.25	0.600
COPD		1.30	0.94	1.78	0.11
Implants/prosthetics		1.13	0.83	1.53	0.451
COVID-19		1.45	0.99	2.12	0.056
Probable primary source of bacteremia	Pneumonia	0.95	0.70	1.30	0.75
	UTI	0.69	0.38	1.24	0.213
	SSI	0.45	0.11	1.83	0.267
	IAI	1.23	0.65	2.34	0.525
	SST	1.39	0.68	2.85	0.364
	Meningitis	0.88	0.32	2.37	0.795
	CVC	0.88	0.54	1.45	0.625
	Endocarditis	2.95	0.73	11.97	0.13
	Spondylodiscitis	2.42	0.60	9.81	0.216
	Unknown source	0.85	0.61	1.21	0.373
Colistin-based treatment		0.71	0.52	0.98	0.037
AKI		1.40	1.02	1.93	0.038
HAI		0.43	0.30	0.61	<0.001

TPN—total parenteral nutrition, COPD—chronic obstructive pulmonary disease, UTI—urinary tract infection, SSI—surgical site infection, IAI—intra-abdominal infection, SST—skin and soft tissue infection, CVC—central venous catheter infection, AKI—acute kidney injury, HAI—hospital-acquired infection.

## Data Availability

Derived data supporting the findings of this study are available from the first author Agnieszka Kuncka upon request.
